# Kallmann Syndrome in a 30‐Year‐Old Female With Primary Infertility: A Case Report

**DOI:** 10.1155/crog/9258447

**Published:** 2026-02-17

**Authors:** Arry Soryadharma, Mulyanusa Amarullah Ritonga, Wiryawan Permadi

**Affiliations:** ^1^ Subdivision of Reproductive Endocrinology and Infertility Division, Department of Obstetrics and Gynecology, Faculty of Medicine, Universitas Padjadjaran–Dr. Hasan Sadikin General Hospital, Bandung, Indonesia, unpad.ac.id

**Keywords:** genetic disorder, Kallmann syndrome, karyotype, primary amenorrhea

## Abstract

Kallmann syndrome (KS) is a rare genetic disorder characterized by hypogonadotropic hypogonadism and anosmia. This case report highlights a 30‐year‐old woman with KS who presented with secondary amenorrhea and uterine hypoplasia after repeated use of combined oral contraceptives (COCs). She had a 5‐year history of primary infertility, hyposmia, and primary amenorrhea. Initially, it was unclear whether her amenorrhea was primary or secondary. However, after interviewing her mother, it was determined that she had primary amenorrhea. Physical examination showed a normal female appearance, with Tanner Stage T1 for pubic hair and T3 for breasts, possibly due to obesity. Hormonal tests revealed low levels of FSH, LH, and estrogen. An MRI of the head demonstrated olfactory bulb aplasia, supporting the clinical diagnosis of KS. Karyotyping confirmed a 46,XX chromosome pattern. The diagnosis of KS was established, and the patient was referred for fertility counseling and ovarian stimulation. KS is typically diagnosed during puberty due to primary amenorrhea, and distinguishing it from other genetic disorders requires karyotyping. This patient′s condition was exacerbated by the repeated use of E‐P pills, which delayed proper diagnosis. Fertility treatment options, including ovarian stimulation with gonadotropins and ovulation induction, were recommended to help the patient conceive. This report emphasizes the importance of careful consideration before administering repeated hormonal treatments and underscores the potential for fertility treatments in females with KS. It highlights the need for clinicians to be vigilant in diagnosing and managing KS, especially in patients with hypogonadotropic hypogonadism and anosmia.

## 1. Introduction

Congenital hypogonadotropic hypogonadism (CHH) is a rare condition characterized by absent or incomplete puberty due to insufficient gonadotropin secretion. CHH includes the anosmic form, Kallmann syndrome (KS), and the normosmic type, idiopathic hypogonadotropic hypogonadism [1]. KS results from defective embryologic migration of gonadotropin‐releasing hormone (GnRH) neurons from the olfactory placode to the hypothalamus, leading to hypogonadism with anosmia or hyposmia. Clinical features vary widely, ranging from absent puberty to spontaneous reversal. Associated anomalies include cleft palate, renal agenesis, dental agenesis, limb defects, and mirror movements [2]. KS prevalence is higher in males (1:30,000) than females (1:125,000), with a Finnish report estimating an incidence of 1:48,000 [3].

KS is genetically heterogeneous, with autosomal dominant, recessive, X‐linked, and oligogenic inheritance reported. Mutations in KAL1, FGFR1, FGF8, PROK2, PROKR2, and WDR11 are implicated, with FGFR1 mutations accounting for ±10% of cases [2, 4].

## 2. Case Report

A 29‐year‐old woman was referred for evaluation of primary infertility associated with uterine hypoplasia and secondary amenorrhea for 1 year. She reported extremely infrequent menses since adolescence, occurring only with hormonal medication, and denied cyclic pain or breast symptoms. She had experienced anosmia since childhood. Menarche occurred at age 18. She had no history of chronic illness, surgery, or contraceptive use.

**Figure 1:**
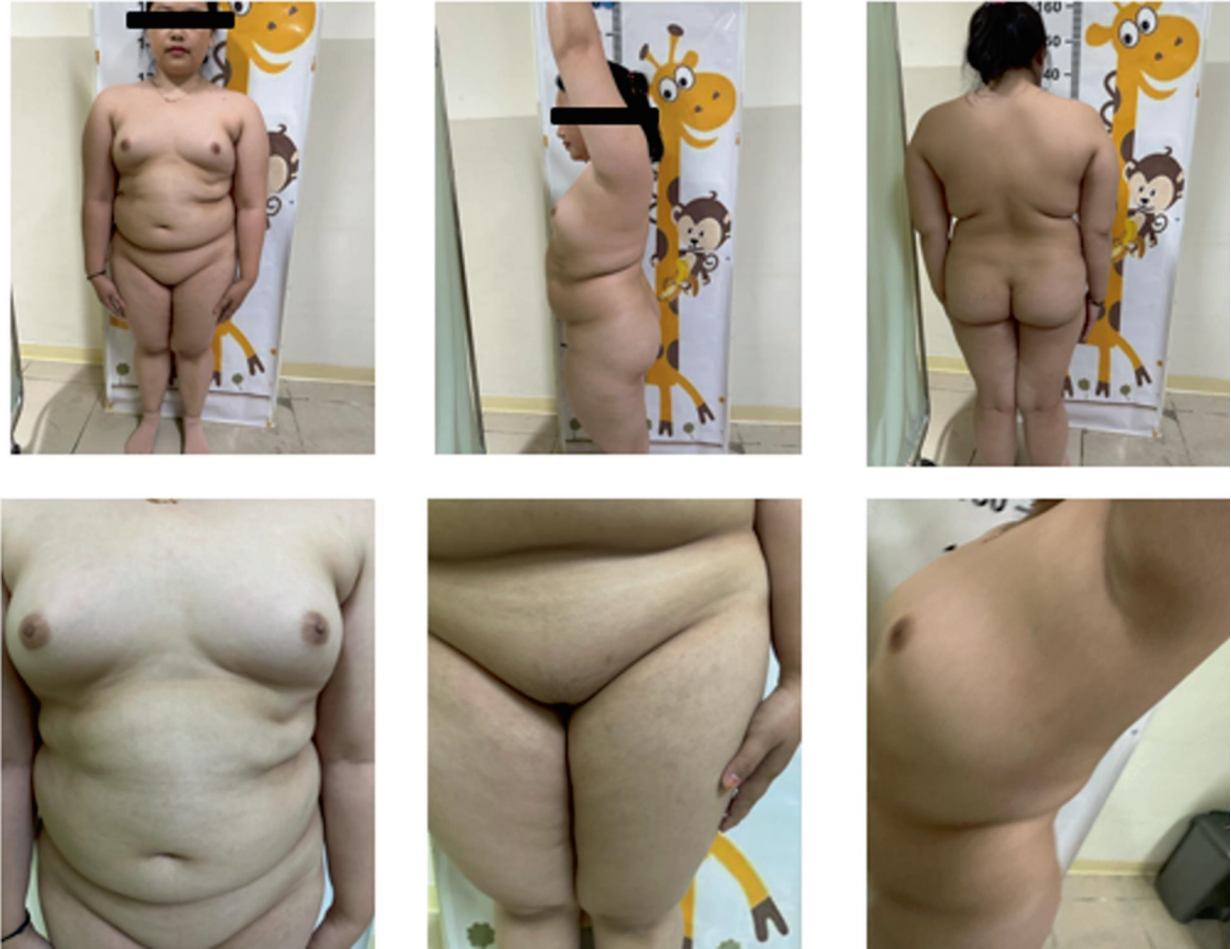
Patient′s physical examination result. The breast development was classified as Tanner Stage T3, without any indication of galactorrhea, and the pubic hair was at Tanner Stage T2.

Examination revealed stable vital signs and obesity (BMI 31.6 kg/m^2^). Secondary sexual characteristics were underdeveloped: breasts Tanner Stage T3 without galactorrhea and pubic hair Tanner Stage T2 (Figure 1). The abdomen was soft and nontender. Internal examination showed a normal vagina and cervix, but the uterus was difficult to assess; adnexa were nonpalpable bilaterally.

**Figure 2:**
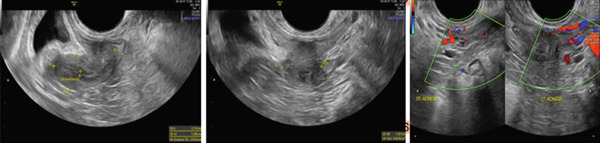
Radiological ultrasound results. Non*cystic* diffuse posterior adenomyosis Type 2–3, heavy, posterior wall thickness 5.53 cm. Uterus anteflexion with homogeneous density, size 4.00 × 2.04 × 1.81 cm, EL 3.63 mm. Bilateral ovaries were difficult to evaluate.

Laboratory evaluation showed negative pregnancy test, low estradiol (< 9 pg/mL), low‐normal gonadotropins (FSH 5.69 mIU/mL, LH 2.86 mIU/mL), normal thyroid function and prolactin, and AMH 1.42 ng/mL. Pelvic ultrasound (Figure 2) demonstrated a hypoplastic uterus (4.00 × 2.04 × 1.81 cm) with thin endometrium (3.63 mm) and Type 2–3 posterior adenomyosis; ovaries were poorly visualized. Karyotype was normal (46,XX).

**Figure 3:**
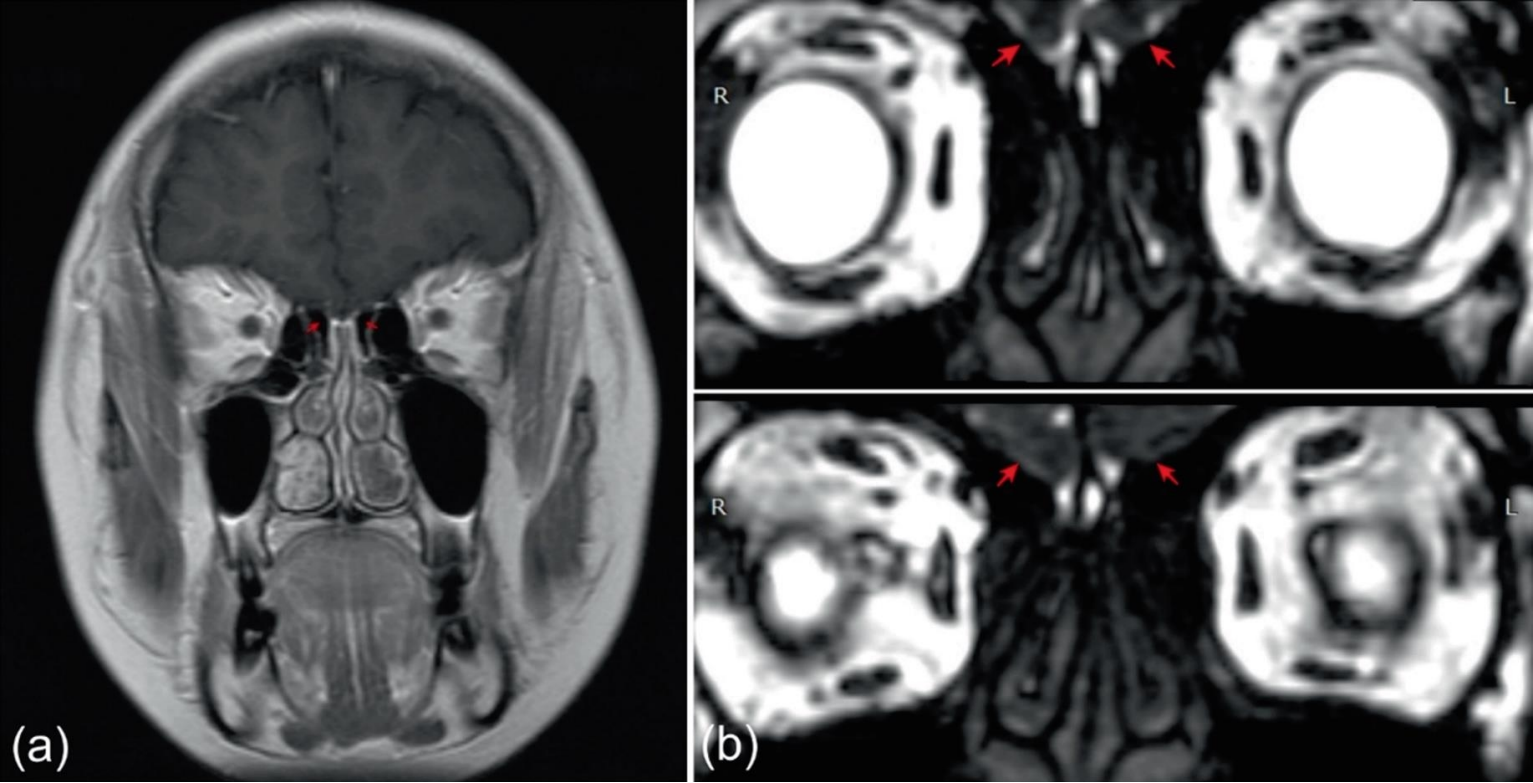
Magnetic resonance imaging (MRI) of the anterior cranial fossa. (a) T1 coronal sequence and (b) CISS sequence. Red arrows indicate the absence of the olfactory bulbs, consistent with olfactory bulb aplasia. The olfactory sulci were only partially captured and could not be reliably evaluated.

Head MRI (Figure 3) using T1 coronal and CISS sequences revealed aplasia of the olfactory bulbs, strongly supporting KS. Although T2 coronal imaging is preferred, available sequences sufficiently demonstrated the characteristic defect. The patient—previously treated only with combined oral contraceptives (COCs) for menstrual induction—showed incomplete breast and uterine maturation typical of women who do not undergo proper pubertal induction with stepwise estradiol therapy. Management included fertility counseling, lifestyle modification, estrogen–progesterone therapy, and one cycle of Cyclo‐Progynova.

## 3. Discussion

KS results from failure of GnRH neuron and olfactory axon migration during embryogenesis [5]. GnRH is essential for stimulation of LH and FSH secretion, which regulate gonadal maturation and reproductive function [4]. After physiologic suppression in childhood, reactivation of pulsatile GnRH initiates puberty, and disruptions at any point along the hypothalamic–pituitary–gonadal (HPG) axis can cause hypogonadotropic hypogonadism.

Over 40 genes have been implicated in CHH/KS, though they explain only half of cases. All inheritance patterns—autosomal dominant (often variably penetrant), recessive, X‐linked, and oligogenic—have been documented, with oligogenic variants accounting for ±15% of cases and contributing to phenotypic variability [4, 6–8].

Defects in genes involved in neuronal migration (KAL1, FGFR1/FGF8, PROK2/PROKR2, NELF, SEMA3A, HS6ST1, CHD7, and WDR11), GnRH secretion (KISS1/KISS1R, TAC3/TACR3, and PCSK1), and gonadotropin regulation (NR5A1, DAX1, and PROP1) can all produce KS [4]. Clinical features vary by age. Adolescents typically present with delayed puberty and absent secondary sexual characteristics. Adult women present with amenorrhea, infertility, low libido, and hypoestrogenic symptoms [9]. Anosmia, although characteristic, is often overlooked in women. Formal olfactory testing helps, as self‐reported normal smell is unreliable. MRI may show absent or hypoplastic olfactory bulbs or sulci, though some anosmic patients have normal imaging [4, 9, 10].

**Figure 4:**
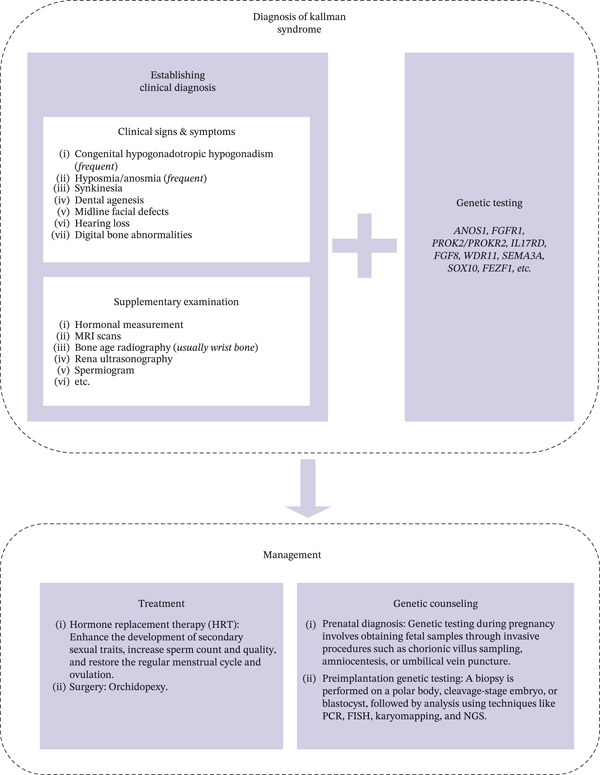
Kallmann syndrome diagnosis algorithm.

Clinical diagnosis of Kallmann syndrome is made by clinical manifestation and supplementary examination. For gold standard diagnosis, genetic testing is needed (Figure 4). In this case, lifelong anosmia, extremely delayed menarche, incomplete pubertal development, low estradiol, and low‐normal gonadotropins strongly indicated central hypogonadism. Uterine hypoplasia, resulting from prolonged estrogen deficiency, is well documented in untreated or inadequately treated CHH [11]. Prior COC therapy—rather than physiologic estradiol titration—likely contributed to suboptimal uterine and breast maturation, similar to other reports [8, 12].

Differential diagnoses include functional hypothalamic amenorrhea, CDGP, CHARGE syndrome, pituitary tumors, and other causes of acquired hypogonadotropic hypogonadism. CDGP is most difficult to distinguish due to overlapping pubertal delay and hormonal profiles [4, 13]. However, anosmia, primary amenorrhea, and olfactory bulb aplasia strongly favored KS in this patient. Karyotyping, though unnecessary in most CHH cases, confirmed 46,XX and excluded Turner syndrome or androgen‐insensitivity syndrome [14].

MRI plays an important role in diagnosis. Olfactory bulb aplasia, as observed here, is a characteristic hallmark recognized since Klingmüller′s early work [15]. Later studies confirmed that absent or hypoplastic bulbs on coronal imaging reliably support KS diagnosis [16].

Management of KS includes pubertal induction and long‐term hormone replacement therapy until at least the age of natural menopause. HRT maintains bone, cardiovascular, and psychosocial health [14]. For fertility, pulsatile GnRH or gonadotropins are first‐line therapy, with IVF reserved as second‐line therapy. Although AMH may appear low in CHH, ovarian reserve is typically normal once gonadotropin stimulation is provided. Pregnancy outcomes are comparable to healthy couples, and HRT should resume postpartum, including during breastfeeding [4, 11, 17].

Although KS does not reduce life expectancy, associated cardiac anomalies, osteoporosis, and infertility may affect long‐term health. Genetic counseling, prenatal diagnosis, and preimplantation genetic testing may be offered due to inheritance risks [3].

## 4. Conclusion

CHH/KS presents with a broad phenotypic spectrum and may closely resemble common reproductive disorders such as CDGP. Early recognition—particularly of anosmia—allows timely treatment, improved pubertal development, fertility outcomes, and long‐term health.

## Funding

No funding was received for this manuscript.

## Disclosure

Each author has indicated that she has met the journal′s requirements for authorship.

## Consent

All the patients allowed personal data processing, and informed consent was obtained from all individual participants included in the study.

## Conflicts of Interest

The authors declare no conflicts of interest.

## Data Availability

The data that support the findings of this study are available from the corresponding author upon reasonable request.
